# Enhancement of microalgal CO_2_ fixation in photobioreactors by means of spiral flow vortices

**DOI:** 10.1186/s13068-025-02650-5

**Published:** 2025-04-29

**Authors:** Santosh Kumar, Ameer Ali Kubar, Xinjuan Hu, Feifei Zhu, Shahid Mehmood, Michael Schagerl, Yajie Zhang, Muhammad Abdur Rehman Shah, Bin Zou, Obaid Ur Rehman, Shuhao Huo

**Affiliations:** 1 School of Food and Biological Engineering, Jiangsu University, Zhenjiang, 212013 China; 2 School of Life Sciences, Jiangsu University, Zhenjiang, 212013 China; 3 Department of Functional and Evolutionary Ecology, University of Vienna, Djerassiplatz 1, 1030 Vienna, Austria; 4 University of Natural Resources and Life Sciences, Vienna (BOKU), Gregor-Mendel-Straße 33, 1180 Vienna, Austria

**Keywords:** Microalgae, Photobioreactor, Portable conical helix baffle, Numerical simulation, CO_2_ fixation

## Abstract

Microalgae have received a lot of interest as a sustainable solution for carbon dioxide fixation due to their great efficiency in capturing CO_2_ and converting it into valuable biomass, making them a promising tool for mitigating climate change and expanding carbon capture technology. This study examines the efficacy of fixed shaped portable conical helix baffles (PCHB) in enhancing gas–liquid mixing to promote microalgal growth in column photobioreactors (PBRs). Flat (90° angle from cone surface), round, and inclined (60° angle from cone surface) baffles were compared for performance. Modeling the gas flow indicated that round PCHB produced more spiral vortices and achieved better mixing performance than flat and inclined designs. Increasing the baffle size from 3 to 7 cm resulted in a 21% higher mass transfer coefficient. The simulation was verified by experiments. Notably, the implementation of a PCHB with a round helix-shaped structure (5 cm) led to a 33% (2.102 ± 0.08 g/L) and 17% (2.419 ± 0.07 g/L) dry mass increase of *Limnospira fusiformis* when compared to flat and incline-shaped baffles, respectively. Our study revealed that using a round-shaped PCHB resulted to higher spiral movement, which in turn increases CO_2_ utilization and cell proliferation. Our approach demonstrates high potential to further optimize industrial PBRs, thereby facilitating CO_2_ sequestration during microalgal cultivation to combat global warming.

## Introduction

The increasing global population growth has fueled an unprecedented demand for energy, leading to heavy reliance on fossil fuels, which constitute approximately 85% of the global energy structure [1]. Despite their pivotal role in powering modern life and enhancing living standards, fossil fuels pose significant environmental challenges due to the emission of greenhouse gases, notably carbon dioxide (CO_2_), exacerbating global climate change [2]. Recognizing the urgency of mitigating CO_2_ emissions and addressing climate change, scientists worldwide are actively exploring renewable energy sources as alternatives to fossil fuels [3]. In this context, microalgal biomass production emerges as a promising solution, offering 2 to 10 times greater CO_2_ fixation than vascular plants [2]. Microalgae use light energy to build up biomass, often with a high nutritional value and a rich source of health-enhancing compounds. Microalgae-based technologies have been tackled as a highly promising avenue for addressing carbon capture in the context of environmental sustainability [4]. Because of their quick growth, microalgae account for around 45% of the global CO_2_ sequestration [5].

Large-scale algal cultivation poses some challenges due to technical complexity associated with the transfer of gases, heat, light, and nutrients [6, 7]. The complex processes involved in microalgae-based CO_2_ fixation and synthesis of bioactive compounds encompass cultivation, harvesting, extraction, and subsequent processing into valuable end products [8]. Microalgae can be grown in either cost-effective open raceway ponds or more productive closed PBRs, which reduce contamination issues. Sustainable and reliable microalgal cultivation within closed PBRs holds pivotal importance for the development of various industries such as biofuels, food, pharmaceuticals and cosmetics [9]. Closed PBRs, including airlift, flat plate, column, horizontal, and tubular designs, are widely employed in microalgal production globally [10]. In comparison to horizontal and tubular design systems, column PBRs (C-PBRs) that incorporate bubble or airlift technologies exhibit better features such as smaller physical footprint, which permits cultivating environments to use their area more effectively [11]. Furthermore, the continual mixing and circulation of culture media made possible by the gas lift or bubble injection leads to better mass transfer and nutrient distribution in column PBRs, which also show reduced fouling tendencies. Since biofilm buildup is reduced in this dynamic environment, maintenance and cleaning procedures are also made simpler. Additionally, modular design of column PBRs improves their scalability by making it simpler to adjust to changing production sizes while preserving operational effectiveness [4]. C-PBRs offer various advantages, including a notable surface area-to-volume ratio, cost-effectiveness in terms of capital investment, absence of moving parts, establishment of a relatively homogeneous culture environment, and mitigation of photoinhibition and photo-oxidation phenomena [12]. The establishment of proficient systems for maximum CO_2_ fixation and oxygen removal however poses a substantial challenge in C-PBRs. Furthermore, efficient mixing and mass transfer setups are imperative to facilitate the stirring up microalgal solutions within C-PBRs.

The integration of baffles in C-PBRs represents a crucial advancement in the field of bioprocess engineering and microalgal-based processes [13]. Previous studies have explored various aspects of the PBR design, yet the specific impact of portable baffle installation in C-PBRs remains a relatively underexplored avenue. Numerous studies focused on increasing the gas–liquid mass transfer, baffle design, microalgal productivity; also, internal lighting with optical fibers inside the C-PBR have been developed to improve the light supply in C-PBRs [14–16]. Ye et al. (2018a) developed the serial lantern PBR which resulted in a 21% reduction in mixing time, a 26% increase of the mass transfer coefficient, a 50% increase in microalgal biomass output, and a greater CO_2_ bio-fixation rate of 0.87 g/L/d. The light supply consists of eight lanterns positioned at the center of the PBR, supported by stainless steel brackets. The fixed baffle structure however limits large-scale applicability by raising cleaning and maintenance costs [17]. Yang et al. (2016) designed unique horizontal tubes and triangular prism baffles, which significantly improved the flashing light effect and raised the production of microalgae biomass by 70%. The triangular prism baffles and horizontal tubes were fixed to the walls to prevent light from entering the PBR's inner part, which makes efficient cleaning a challenge [18]. Naira et al. (2020) designed bubble-driven internal mixers which increased the biodiesel and biomass productivity by 62% and 13%, respectively, in comparison to controls run without mixers. Also, this approach is applicable to limited extent since the complicated mixer structure generates flow resistance and the movement of wings at some distance from bubble source was not clarified [19]. Cheng et al. (2018) developed a double paddle wheeled PBR, resulting in a reduction of average bubble size by 24% and a decrease in bubble vertical velocity by 10%. These alterations enabled enhanced mixing of CO_2_ and mass transfer within the microalgal solution. Consequently, growth rate of *Chlorella* sp. in this C-PBR exhibited a notable increase of 62% with 15% CO_2_ aeration. The problem was however that biomass was deposited between the blades of paddle wheel [20]. Fu et al. (2021) designed airfoil-shaped deflectors that modified hydrodynamic parameters. By this means, the mass transfer coefficient increased by 11% and the mixing time decreased by 21%. The maximum yield of algal biomass and CO_2_ fixation improved by 18% and 11%, respectively. Nevertheless, rapid gas escape occurred due to the limited effects observed in the bottom and middle sections of the C-PBR, which consequently reduced the efficiency of CO_2_ fixation [21]. However, most of the studies have a fixed design and complex structures of baffles which limit their application in C-PBRs. This portable baffle design is a novel advancement in C-PBRs, as it has not been utilized before. Its innovative features facilitate easy operation and cleaning, significantly enhancing the efficiency of microalgal cultivation processes.

In this study, 3D-printed portable conical helix baffle (PCHB) with different sizes were installed in the center of 0.08 m wide and 0.52 m long inner section of C-PBRs to generate spiral flow vortices. The introduction of a PCHB in C-PBR resulted in spiral-rising CO_2_ bubble flow patterns, as validated by numerical simulation. Furthermore, experiments focusing on mixing time and mass transfer showed that using the PCHB improved mass transfer and bubble retention time, which subsequently raised microalgal growth, photochemical efficiency and pigment concentration. The baffles shape, size and aeration rate were optimized to enhance the overall efficiency and productivity of microalgal cultivation practices.

## Materials and methods

### Simulation of C-PBR modified with portable conical helix baffle

To enhance the performance of C-PBRs, PCHBs were utilized to increase the number of vortices in the culture medium. The C-PBR was divided into two sections: a central column (riser section) and an outer column (downcomer section), with diameters of 0.08 m and 0.12 m, and heights of 0.52 m and 0.60 m, respectively. The PCHB baffles underwent design modifications with diverse parameters utilizing Rhino-7 (3D design software), subsequently being fabricated through 3D printing utilizing nylon material [22]. Nylon is durable, flexible, and resistant to chemicals, which are essential characteristics for C-PBR environments. Nylon is typically non-toxic to microorganisms and regarded as biocompatible, as it is frequently utilized in medical devices and implants, including sutures and prosthetics. Ansys-21 ICEM (Ansys, 64-bit Computational Fluid Dynamics, CFD software, USA) was used to create a two-dimensional mesh of the C-PBR. A CFD simulation was then run using Ansys Fluent-21 R1. Boundary conditions of the numerical model were as follows: both the PBR walls and the channels were configured with no-slip wall conditions, and the gas outlet was positioned in relation to the degassing boundary. The RNG k-ɛ turbulence model and the volume of fluid (VOF) model were used in the computational simulation. The time step number was set to 10,000 and the transient time step size was set to 0.002 s. To ensure grid independence, three-scale grids 13,835, 17,942, and 22,471 cells were taken. As we found only marginal variations in the computed values, we used the mesh of 17,942 cells for all situations [23]. The C-PBR's overall air-volume and velocity contours were measured, and the computation was carried out twice in quick succession on different machines for verification.

### Experiments for solution mixing and determination of the mass transfer coefficient

**Fig. 1:**
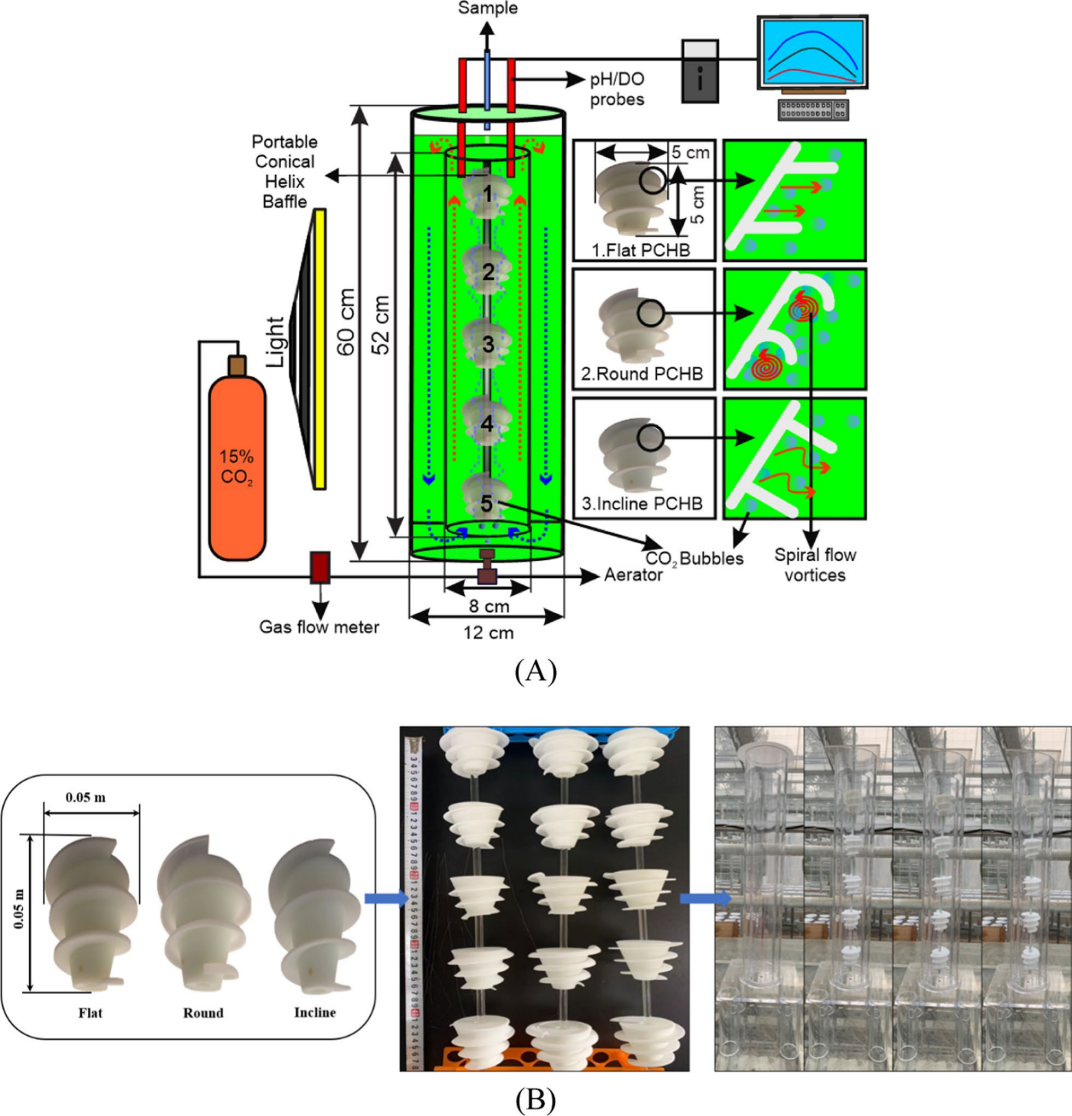
Schematic of column PBR and installation of portable conical helix baffles in central column sections. A Five round-shaped baffles connected with a supporting rod in vertical position. Three different shapes of helix: flat, round, and inclined, and their respective flow vortices represented in the side small boxes. Red-dotted arrows represent upward flow, blue-dotted arrows represent downward flow of the medium. B Three-dimensional printed 3 types of portable conical helix baffles, their vertical arrangement and installation in lab-scale column PBRs. (For elucidation regarding the color references in this figure legend, readers are directed to consult the online version of this article.)

To enhance the C-PBR design, various PCHB baffle sizes, ranging from 0.03 to 0.07 m, were designed. Additionally, three different baffle shapes were chosen to aid in the optimization process. These variations are flat (90° angle from cone surface), round, and an incline-shaped (60° angle from cone surface) baffles as shown in Fig. 1 A. Four lab-scale C-PBRs were constructed with 3 mm thick plexiglass, each with 3D-printed PCHB (fixed shaped structures) placed in the inner section vertically connected with a supportive rod holding baffles at 0.05 m equal intervals in the center (Fig. 1 B). The mixing time defined as a period of time required to achieve homogeneous mixing of a fluid in a container, or the time necessary for a tracer to achieve 95% uniform distribution in the liquid after its introduction. To measure the mixing time in the PBRs, a pH probe was fixed at the top of the central column (length 0.52 m) and a tracer was introduced from top of the outer column (length 0.60 m) as shown in Fig. 1A. The C-PBRs were initially filled with tap water and the bubbling started. The pH of the water was adjusted to around 3.0 using hydrochloric acid (35% w/v). Subsequently, an alkalinity tracer consisting of 5 mL of 12 M sodium hydroxide was introduced into the medium. The mixing time was determined by the interval between the adding the alkalinity tracer and measuring the pH shift [24]. The mass transfer coefficient was determined by aerating the solution with N_2_, resulting in a reduction of oxygen content to 4 mg/L. Subsequently, air was introduced to elevate the dissolved oxygen (DO) content to 5 mg/L. Data were recorded automatically at intervals of 0.1 s using an oxygen probe; data acquisition was conducted through dedicated software [25]. Means and standard deviation of mixing time and mass transfer coefficient were calculated based on three (n = 3) independent measurements.

### Experimental verification—cultivation of Limnospira fusiformis

Cultivation was conducted in four C-PBRs, each consisting of inner and outer columns measuring 0.60 m and 0.52 m in length, respectively, with diameters of 0.12 m and 0.08 m. The experiment was conducted three times (n = 3). In each C-PBR, five identical (either flat, round or incline-shaped) PCHBs with 0.05 m size were installed, one C-PBR was without PCHBs to serve as a control for comparison. We cultivated the cyanoprokaryote *Limnospira fusiformis* (tradename *Spirulina platensis*), which is of high commercial interest; cultivation was done in Zarrouk medium [26]. The experiments were carried out in an artificial greenhouse (for 120 h), maintaining a constant temperature of 28 ± 1 °C by utilizing a 15-W digital quartz electric heating rod (diameter 0.02 m, length 0.06 m, temperature control range: 20–35 °C) within each C-PBR. In order to maximize solution mixing and gas–liquid mass transfer to produce higher biomass, the dimensions of PCHBs were thoroughly complied. Installation of the PCHB markedly diverged the bubble trajectory, thereby augmenting the mixing and dissolution of CO_2_ along the baffles as they ascended beyond the surface. Additionally, a gas mixture containing 15% CO_2_ and 85% air was introduced through aeration from the lower base-center. The CO_2_ fixation rate was measured via the dry weight conversion method using following equation:

$${CO}_{2 }fixation \left(g{L}^{-1}{d}^{-1}\right)=\frac{\left({w}_{2}- {w}_{1}\right)\times P\times {M}_{CO2} }{\left({t}_{2}-{t}_{1}\right) \times {M}_{c}},$$

where w_2_ is the biomass dry weight at time t_2_ and w_1_ is the biomass dry weight at time t_1_, p represents carbon content in microalgae cells (50%), M_CO2_ represents the molecular weight of CO_2_, and M_C_ denotes the molecular weight of carbon [27–29].

Using 15% CO_2_ provides a regulated environment to promote growth while avoiding significant changes in solution pH. The gas flow rate was consistently set at 0.003 L/min through the use of mass flowmeters. The cultures were grown under a 12-h light/dark cycle (300 µmol/m^2^/s) with bright LED lamps providing a light intensity of 5000 ± 200 lx. Every day at 9:00 during batch cultivation, samples were collected in order to measure pH, optical density (OD), biomass, maximum quantum efficiency of PSII (Fv/Fm), and pigments. A total of 0.01 L of sample was collected to measure the culture's OD and pH (LIDA pH meter). The relationship between OD and cell density (C) was quantified by the fitting curve, represented as $C\left(\frac{g}{L}\right)=0.505 \times {OD}_{560 nm}-0.034$, with a coefficient of determination (R^2^) of 0.996 [30].

The concentrations of carotenoids and chlorophyll-a in microalgal cultures were determined over the course of the culture. An 8-min centrifugation at 8,000 × g at room temperature was performed on 0.005 L of the collected microalgal culture; the supernatant was discarded. After being chilled to 4 °C, 0.005 L of methanol is added to the microalgal pellet. To determine the concentrations of carotenoids (480 nm) and chlorophyll-a (652 nm and 665 nm) a spectrophotometer (752-N Youke Instrument, Shanghai, China) was used to measure absorbance at 480 nm, 652 nm, and 665 nm, with pure methanol used as the blank [31].

In order to extract allophycocyanin and phycocyanin, the culture was centrifuged at 5000 × g for 4 min. After that, the supernatant was removed and the pellet mixed for 2 min with a 0.005 L solution of CaCl_2_ (1% calcium chloride). These solutions were frozen and then thawed three times before being incubated at 4 °C for the entire night [32]. The absorption value of supernatant was determined using a spectrophotometer at wavelengths of 652 nm for allophycocyanin and 620 nm for phycocyanin subsequent to centrifugation of the sample for 12 min at a rate of 8000 × *g*. All measurements were repeated at least three times (*n* = 3). The mean value and standard deviation were taken for statistical comparisons.

## Results and discussion

### PCHB baffles generate spiral flow field in the C-PBRs

The increase in hydraulic pressure associated with larger bubble diameters, as described by the buoyancy lift force equation and the Young–Laplace equation, facilitates the ascent of CO_2_-enriched bubbles [33]. Consequently, bubbles spend less time in the microalgal suspension at higher gas aeration rates [34]. Introducing a PCHB significantly alters the flow pattern and trajectory of bubbles within the C-PBRs. Unlike in control system where bubbles detach and ascend directly to the surface, the PCHB encourage bubble breakage, with liquid drag playing a dominant role in their movement. This force bubbles to circulate multiple times around the PCHB before reaching the top of C-PBR, significantly prolonging their pathway and enhancing gas–liquid contact time, thereby promoting CO_2_ dissolution. Moreover, reduced bubble size has a greater specific surface area and interfacial contact area between gas and liquid phases, thereby enhancing the performance of gas–liquid mass transfer [35]. Smaller bubble diameters correspond to elevated CO_2_ equilibrium concentrations between the gas and liquid phases. This phenomenon can be attributed to the rapid absorption of CO_2_ by microalgal cells, resulting in an augmented CO_2_ concentration gradient, enhanced diffusion and dissolution of CO_2_ bubbles [36].

There is not enough gas–liquid mixing in the C-PBRs for adequate CO_2_ mass transfer, leading to an uneven distribution of nutrients and cells [37]. In order to enhance microalgal biomass production, agitation is required to sustain a stable microalgal culture to circulate fluids. Mixing serves to prevent the sedimentation of cells on baffles and along the sides as well as on walls of the PBR, also facilitating the removal of oxygen from the culture medium [38]. Additionally, shear stress, induced by baffles, represents a significant parameter during the circulation of the culture. Shear stress, which is produced by mixing, is considered significant as it can facilitate mass and heat transmission as well as the flashlight effect to augment microalgal photosynthetic activities [39]. The preceding discourse indicates that cellular rotation induced by mixing may enhance the surface area exposed to illumination, ensure uniform solution temperature, and increase the rate of CO_2_ fixation, facilitated by the flow vortices along the PCHB.

**Fig. 2:**
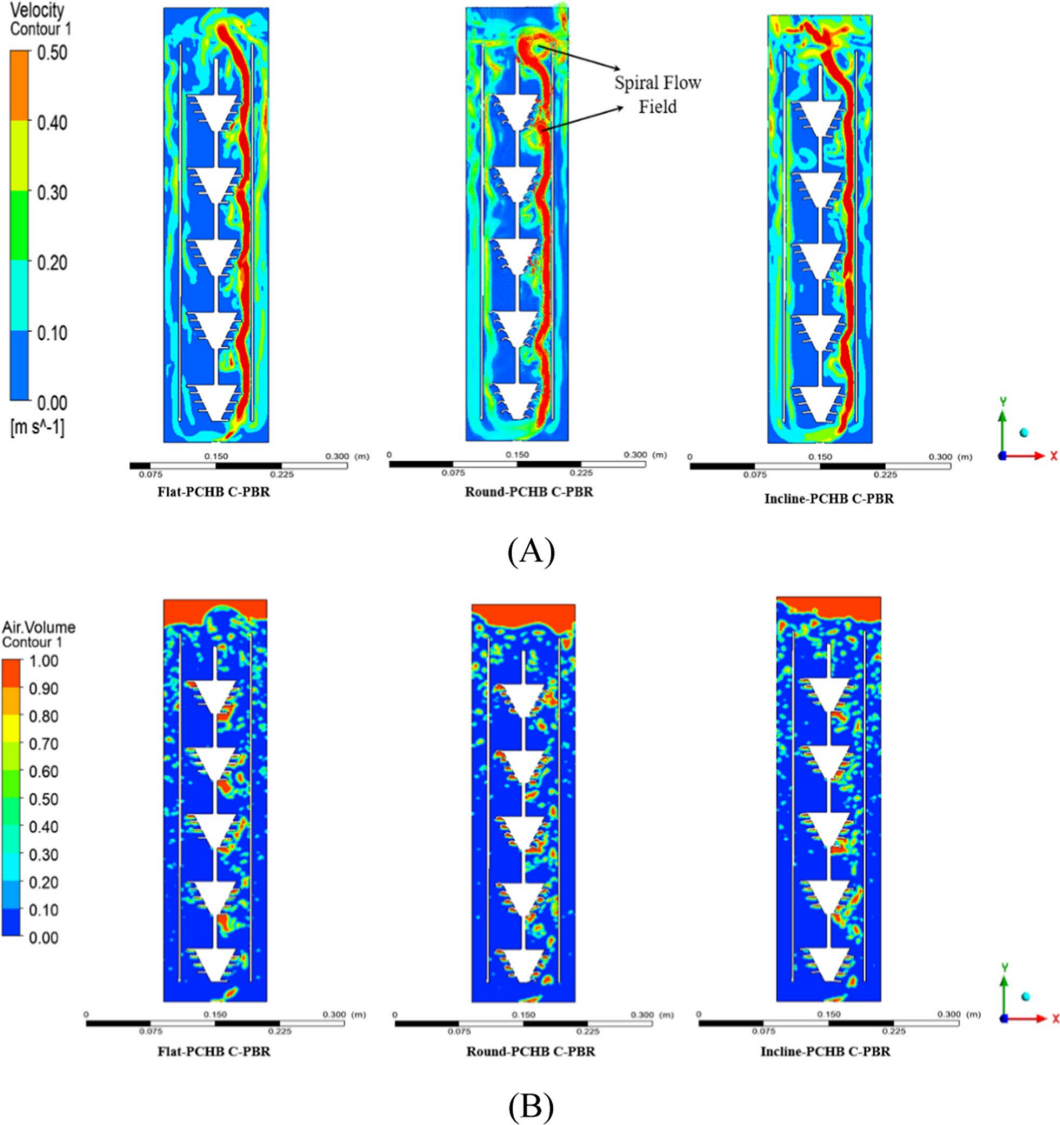
CFD simulation of flat, round and incline portable conical helix baffles in column PBR. **A** Velocity counter illustration. **B** Air. Volume contour illustration

In a C-PBR, PCHB produced a rotational and spiral flow vortex along the central inner section as shown in Fig. 2 A, B. Both vortexes along the PCHB were indicated by CFD simulation calculation. The creation of three-dimensional flow patterns by a submerged object within a uniform free-flowing medium of limited depth induces shear stress [39]. The bubble drive to axially directed spiral motion in x, y, and z directions was essential for gas–liquid mixing, and it significantly extended the bubble residence time [40]. Liquid mixing and bubble residence are enhanced by the creation of rotational and spiral flow vortex and the improvement of vorticity magnitude and horizontal velocity in the horizontal direction, which promotes microalgal productivity [34]. Simulation results clearly indicated that PCHB encourage bubble spiral flow movement on the vertical cross section along the main stream. This implies that the PCHB has the capability to generate a spiral flow field around baffles in the central inner column section. Increasing the vorticity magnitude and vertical velocity, coupled with the creation of spiral flow vortices, increase mass transfer coefficient and bubble retention, consequently stimulating microalgal growth. The present work employed 2D simulations to model impacts of different PCHB types. The 2D simulations provided a simple approximation to the most efficient type. For fine adjustment of PCHBs, 3D simulations will be performed to further optimize the design. 3D simulation will help modeling important factors such as bubble size, flow patterns, CO_2_ movement, gas–liquid interaction, and CO_2_ fixation rates.

### Improved bubble mixing and mass transfer by means of PCHB

**Fig. 3:**
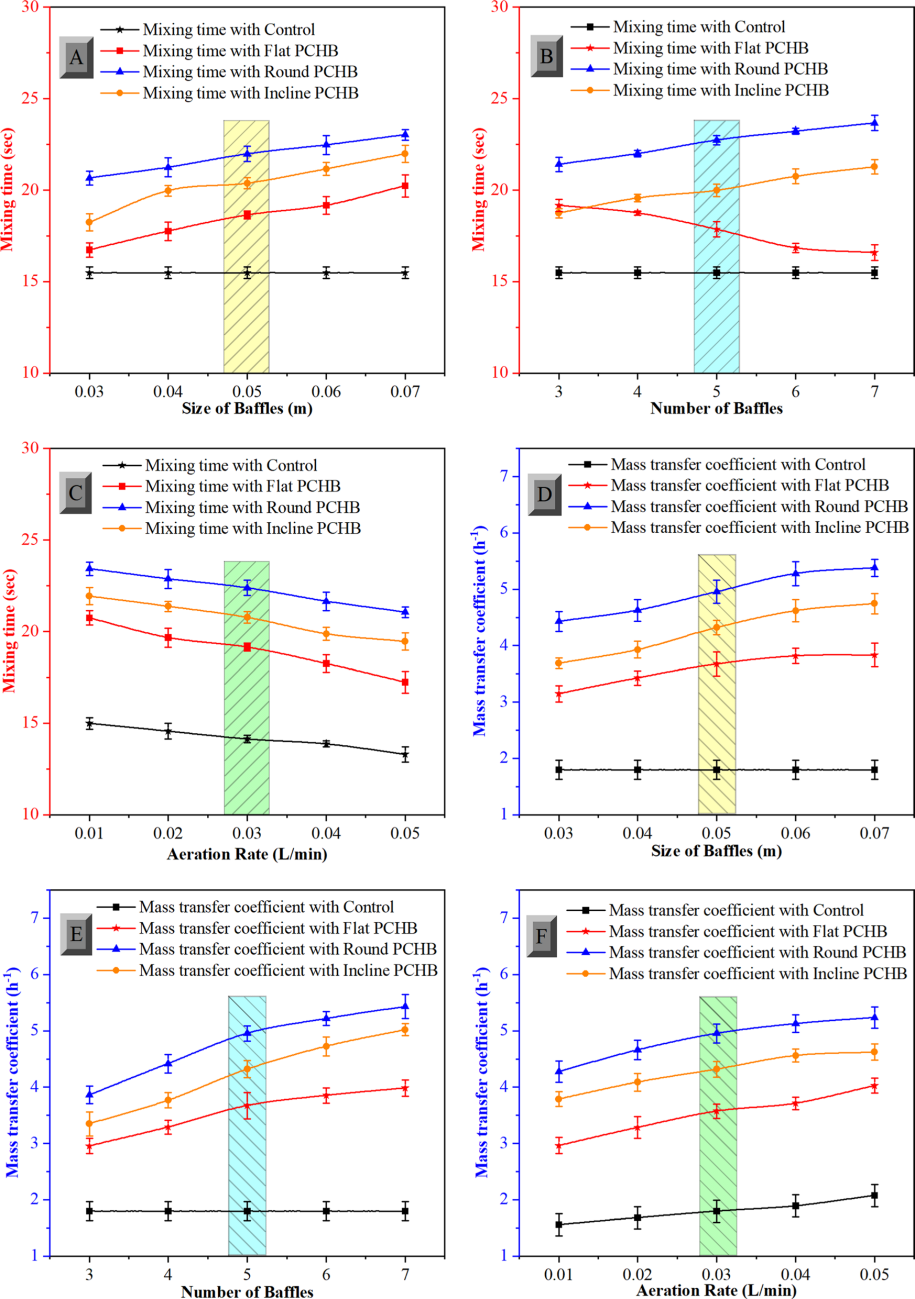
Effects of portable conical helix baffles on mixing time and mass transfer coefficient in the column PBRs. **A** Effects of size of baffles on mixing time. **B** Effects of number of baffles on mixing time. **C** Effects of aeration rate on mixing time. **D** Effects of size of baffles on mass transfer coefficient. E Effects of number of baffles on mass transfer coefficient. F Effects of aeration rate on mass transfer coefficient. Note: The mixing time was 15.48 s and mass transfer coefficient was 1.79 per hour in traditional column PBR unmodified with baffles

In order to optimize the C-PBR, we modified the shape of PCHB. Additionally, the size, number of baffles and aeration rate were the three main parameters to be investigated to enhance the performance of the control C-PBR. The relationship between bubble residence time and mass transfer coefficients is acknowledged, revealing that prolonged bubble residence in cultures contribute to elevated mass transfer coefficients, thereby fostering favorable conditions for microalgae growth [24]. We found an extension in the mixing time of liquids due to the addition of PCHB, leading to enhanced nutrient supply. Mixing time increased by 42%, 46%, 57% with flat, incline and round PCHB when compared with control PBR under optimized conditions as resulted in Fig. 3A–C, respectively. The results demonstrate an increase in mixing time from 20.65 ± 0.3 to 23.02 ± 0.2 s with an increase in the size of PCHB from 0.03 to 0.07 m and increase from 21.40 ± 0.3 to 23.67 ± 0.4 s with an increase the number of PCHB from 3 to 7 as resulted in Fig. 3A, B. This phenomenon is attributed to the larger PCHB size hindering the normal upward flow of the CO_2_ bubbles from the bottom to the surface. The larger size induces higher resistance in the flow, facilitating the generation of vortices and spiral movements in both horizontal and vertical directions along the PCHB, restricting a direct flow to the top surface within the C-PBR. The aeration rate raised from 0.01 to 0.05 L/min; the mixing time reduced from 23.42 ± 0.4 to 21 ± 0.1 s as shown in Fig. 3C. The enhanced aeration rate increased the microalgal suspension circulation process, shortening the mixing period and increasing flow intensity, thus reducing mixing time [17].

The rotational and spiral flow field by the PCHB is the primary factor that determines the inner mixing ability of the C-PBR. The microalgal suspension is directed through PCHB baffles within the C-PBR configuration, thereby extending the flow trajectory. This extension enhances the mixing dynamics and promotes a homogeneous distribution of cells within microalgal suspensions, facilitating vertical movement across central and peripheral regions within the column [16]. Conversely, the control C-PBR demonstrates reduced mixing efficiency attributable to the presence of relatively wide inactive zones. In such areas, substance exchange predominantly relies on diffusion, thereby markedly impeding the mixing kinetics [37].

To attain greater biomass output while cultivating *L. fusiformis*, the mass transfer coefficient is an essential measure that determines the baffle design within the C-PBR. An increase in the gas–liquid mass transfer coefficient was observed, leading to enhanced CO_2_ uptake by microalgal cells. Larger diameters of PCHB from 0.03 m to 0.07 m led to a higher mass transfer coefficient, as depicted in Fig. 3 D, E, F. Larger PCHBs resulted in greater shear strength to the CO_2_ bubbles, rising liquid mixing and prolonging bubble residence. This phenomenon facilitated the formation of flow vortex and the enhancement of vorticity magnitude and vertical velocity, ultimately resulting in higher microalgal growth through improved solution mixing [36]. In the optimized PCHB, a high mass transfer coefficient of 4.95 ± 0.1 h^−1^ was observed when the size of the baffle was 0.05 m and the aeration rate was 0.03 L/min, as shown in Fig. 3 E. In this scenario, optimal bubble movement was achieved, and the flow path was tailored to facilitate effective solubility of CO_2_. Further increasing bubble retention time will no longer have a positive influence on CO_2_ transfer, while the negative effect of increased flow resistance induced by the larger bubble size combined with high aeration rate becomes the primary factor thus reducing the mass transfer coefficient. C-PBR characterized by a high mass transfer capacity retain substantial quantities of inorganic carbon within the column, thereby boosting facilitating microalgal fixation and consequentially nurturing robust microalgal production [25]. Furthermore, C-PBR with high mass transfer coefficients effectively remove accumulated dissolved oxygen, reducing oxygen-induced stress and maintaining a high rate of microalgal growth. It is understood that the formation of spiral flow vortices, improvements in mass transfer coefficient, optimize mixing time, and increased bubble retention collectively enhance the microalgal growth.

For optimal biomass production of *L. fusiformis*, the gas–liquid mass transfer coefficient is key in determining the baffle design within a column photobioreactor (C-PBR). Larger diameters of portable conical helix baffles (PCHB) ranging from 0.03 m to 0.07 m increased CO_2_ uptake by promoting better liquid mixing and prolonged bubble retention. This generated spiral flow vortices and higher vertical velocity, improving microalgal growth. However, excessive bubble retention increased resistance, lowering mass transfer efficiency.

### Microalgal growth and photosynthetic fluorescence improved with PCHB

**Fig. 4:**
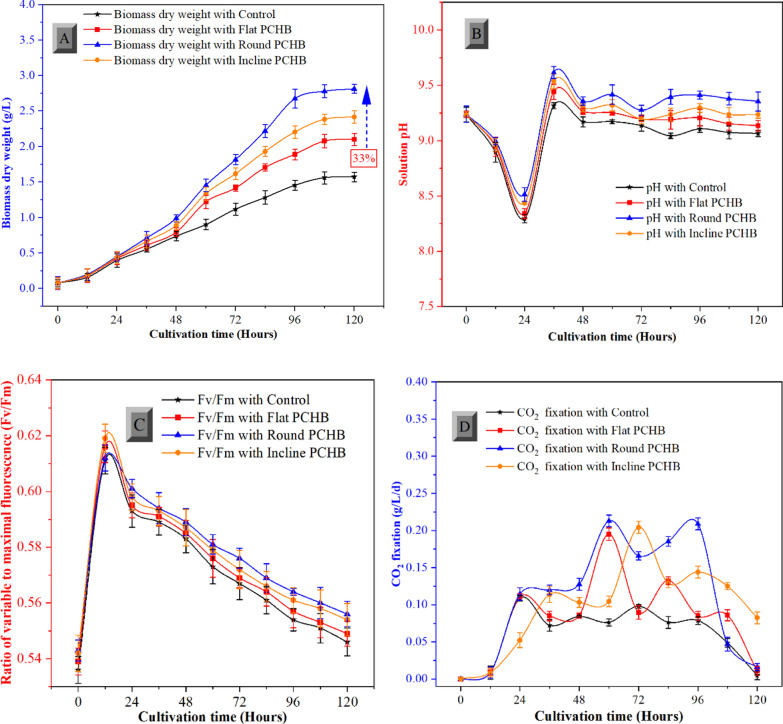
Measurement of biomass, pH, ratio of variable to maximal fluorescence (Fv/Fm) and CO_2_ fixation of microalgae in column PBRs modified with portable conical helix baffles (PCHBs). **A** Effects of PCHBs on dry mass of *Limnospira fusiformis*. **B** Measurement of solution pH during cultivation of *Limnospira*. **C** Effects of PCHBs on maximum quantum efficiency of PSII (Fv/Fm) of *Limnospira*. **D** Effects of PCHBs on CO2 fixation rate during cultivation of *Limnospira*

The preceding literature demonstrates a direct correlation between the CO_2_ supply and productivity. Specifically, an increase in the utilization of CO_2_ corresponds to a commensurate rise in biomass growth [34]. We measured the dry mass for three differently shaped PCHB and one control C-PBR without PCHB installed. We found the characteristic shape of a growth curve with a lag-phase (acclimation phase) characterized by reduced growth rates, after around 36 h, the exp-phase started with maximum growth rates. C-PBRs with PCHB installed exhibited higher growth rates (Fig. 4A) due to appropriate mixing and optimized CO_2_ supply. Effective nutrient uptake is imperative for the proliferation of microalgal cells [21]. The spiral flow vortices by means of PCHB maintain homogenous distribution of cells and nutrients and enhance the efficiency of chemical conversion in microalgae. The exp-phase lasted for about 60 h and resulted a higher biomass growth from the modified C-PBRs compared to the control. After 96 h, algae growth slowed down, which indicated the transition to the stat-phase. The experiment was finished after 120 h: round-shaped PCHB resulted in a 48%, 34% and 16% higher biomass yield comparison to control, flat and inclined-shaped PCHB, respectively.

Several studies have focused on various baffle designs to enhance microalgal biomass production in C-PBRs. Huang et al. (2015) introduced inclined plate mixers in the outer section of the C-PBRs resulting an increase in biomass concentration by 32.8% (0.89 g/L) [41]. Ye et al. (2018b) focusing on wastewater as a medium, proposed a serial lantern-shaped inner structure, which improved mixing and mass transfer, leading to a 50% (1.8 g/L) increase in biomass concentration compared to controls [23]. Fu et al. (2021) installed two airfoil-shaped arcs at the top of the C-PBR, resulting in an 18.3% (2.1 g/L) rise in biomass content [42]. Similarly, Yang et al. (2016) designed circular baffles at the center along with a prism structure at the wall, significantly boosting biomass yield by 70% (1.2 g/L) [43]. These studies highlight the effectiveness of various structural modifications in improving biomass productivity. Similarly, the current study with PCHBs in the inner section of the C-PBRs resulted in an 48% increase (2.81 ± 0.06 g/L) in dry mass.

shows that pH decreased initially and increased after 24 h of cultivation. The primary chemical reaction in the microalgal solution during the first stage of cultivation was the mixing of CO_2_ with water, producing bicarbonate ions (HCO_3_^−^) and hydrogen ions (H^+^). This caused the pH to decrease from 9.23 ± 0.06 to 8.51 ± 0.07, as more H^+^ ions were produced. As *L. fusiformis* grew, the HCO_3_^−^ in the solution were absorbed by cells through active transport. Inside the cells, HCO_3_^−^ was converted to CO_2_ and used in photosynthesis, during which hydroxide ions (OH^−^) were expelled from *Limnospira* cells. This led to a gradual increase in the pH of the solution, which rose to around 9.61 ± 0.05

Significant amounts of photochemical energy, particularly ATP and NADPH, are required by microalgal cells for biomass synthesis, CO_2_ fixation, and active transport mechanisms during cellular growth [44]. In the context of *L. fusiformis* cultivation, improvements to the PCHB setup within the C-PBR resulted in improved solution mixing. In comparison to control C-PBR, the round-shaped PCHB showed an 18% increase in the ratio of variable to maximal fluorescence (Fv/Fm) as resulted in Fig. 4C. The overall photosynthetic performance, as indicated by the Fv/Fm ratio, may show lower values due to interference of the fluorescence signal by phycobilin. A reduced Fv/Fm ratio may not always indicate stress; it can also result from increased phycobilin synthesis within *L. fusiformis* cells [45]. Adequate mixing facilitated by PCHBs facilitated improved light distribution to the cells. Initially, the Fv/Fm ratio exhibited an ascent followed by a gradual decline after the 12 h of cultivation, attributable to higher cell density within the C-PBRs, wherein the increasing biomass dry weight rendered the solution denser. Oversaturated *Limnospira* cell densities generated a mutual shadowing effect, reducing light use ability. Consequently, increased fluorescence was required to achieve uniform photosynthetic activity. The overall biomass production was improved by the higher Fv/Fm values, which also led to improved light utilization capacity per photon received by the cells and enhanced photochemical efficiency. *Limnospira* cells accelerated photosynthetic reaction was made possible by the different vortex flow fields created throughout the C-PBR [46]. Additionally, the increased flashing-light frequency accelerated microalgal cell growth [47]. The enhanced spiral flow and elevated vertical movement facilitated the migration of microalgal cells from the central region, characterized by reduced illumination, to the periphery of the round-shaped PCHB. This quick movement between areas of light and dark greatly decreased the likelihood of photodamage or low light in microalgal cells [42]. The PCHB improved flow field which promote microalgal growth rate, leading to accelerated pigment content and higher photosynthetic rates compared to control C-BPR.

The PCHB C-PBRs demonstrated a higher rate of CO_2_ fixation than the control C-PBR, which is in line with a higher level of CO_2_ fixation by *L. fusiformis*, as illustrated in Fig. 4D. The round-shaped PCHB allowed for efficient mixing throughout the C-PBR, which improved the pace at which CO_2_ was fixed. After 72 h, the CO_2_ fixation rate of *L. fusiformis* peaked at 0.212 ± 0.005 g/L in the C-PBR with round-shaped PCHB and 0.097 ± 0.002 g/L in the control C-PBR. The CO_2_ fixation rate of *L. fusiformis* in flat and incline-shaped C-PBRs were also higher than control C-PBR and peaked at 0.195 ± 0.008 g/L and 0.204 ± 0.006 g/L, respectively. However, at 120 h, the CO_2_ fixation efficiency declined as the growth rate of *L. fusiformis* increased, attributed to the depletion of nutrients in the culture medium.

### Pigment content stimulated by the PCHB in the C-PBR

**Fig. 5:**
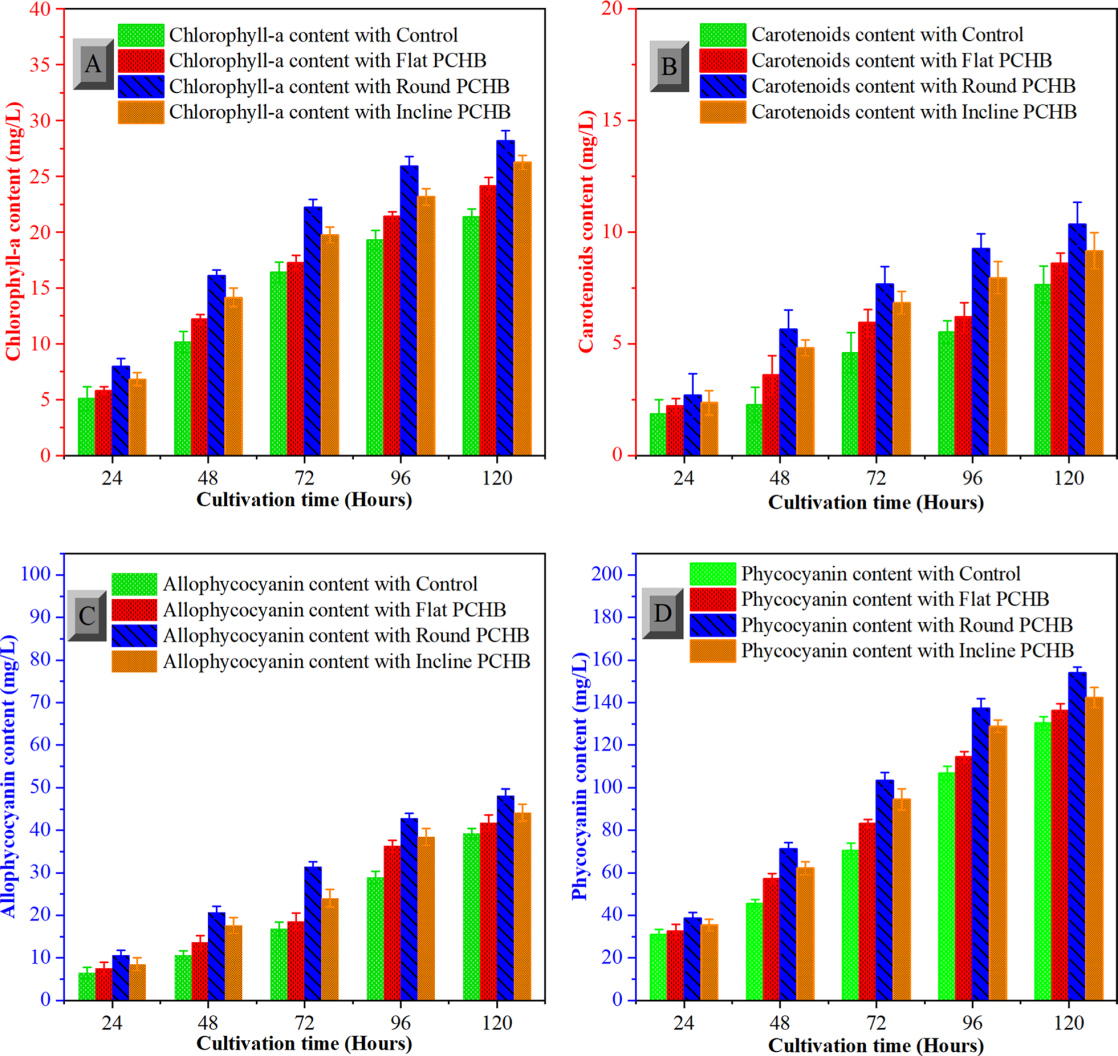
Measurement of cellular pigment content of microalgae in column PBRs modified with portable conical helix baffles. **A** Effects of portable conical helix baffles on cellular chlorophyll-a content. **B** Effects of portable conical helix baffles on cellular carotenoids content. **C** Effects of portable conical helix baffles on cellular allophycocyanin content. **D** Effects of portable conical helix baffles on cellular phycocyanin content of *Limnospira fusiformis*

The carotenoids and chlorophyll-a concentrations were considerably greater in PCHB-cultured cells compared to control C-PBRs, as illustrated in Fig. 5A, B. The photosynthetic apparatus underwent acclimatization in response to variations in light spectra, resulting in adjustments in the abundance of accessory pigments and the chlorophyll-a content. Chlorophyll a, a crucial pigment in organisms performing oxygenic photosynthesis, plays a vital role in photosynthesis by enabling cells to capture light energy and convert it into ATP [48]. Higher cellular concentrations of chlorophylls in plant cells enhance light utilization efficiency. In cultures employing round-shaped PCHB, chlorophyll-a concentrations were notably elevated by 31% (28.19 ± 0.9 mg/L) when compared with control (21.36 ± 0.6 mg/L), highlighting the positive impact of improved mixing. The synthesis of chlorophyll-a demands substantial energy, primarily derived from incident light photons and stored in microalgal cells. Among other factors, provided light conditions significantly influence ATP production, thereby affecting chlorophyll synthesis [49]. The optimized vertical helix arrangement of the PCHB in C-PBRs enhanced light absorption efficiency, which increased biochemical energy production during the light reactions and resulted in higher average chlorophyll content. However, the influence of inclined and flat-shaped PCHB on chlorophyll-a synthesis was limited due to insufficient mixing and extensive shaded area coverage. The carotenoids in *Limnospira* cells were also higher in round-shaped PCHB (10.36 ± 0.9 mg/L) when compared with control (7.63 ± 0.8 mg/L) C-PBR as resulted in Fig. 5B.

In the realm of commercial potential, *L. fusiformis*, particularly its phycocyanin pigment, holds significant potential [32]. The market valuation of C-phycocyanin may vary between US$500 and 10,000 per kilogram, contingent upon its degree of purity [50]. Increasing the biomass yield of *L. fusiformis* presents a prospective avenue for mitigating production expenses and bolstering market competitiveness. It is essential to optimize light supply to *Limnospira* filaments to boost productivity. Control C-PBRs frequently demonstrate restricted vertical and turbulent mixing within the central portion of the column, resulting in extensive areas of laminar flow throughout the column, wherein microalgae are deprived of illumination [51]. Cells located near the center of column receive insufficient illumination while cells along the walls experience excess light resulting in photoinhibition, which has negative effects on productivity [52]. A higher concentration of allophycocyanin and phycocyanin was observed in samples from the round-shaped PCHB C-PBR, with the lowest concentrations measured in the control C-PBR without PCHB. After 120 h of cultivation, the phycocyanin concentration in the round-shaped PCHB C-PBR was 154.17 ± 2.6 mg/L, while it reached 130.34 ± 2.9 mg/L in the control PBR, as shown in Fig. 5C. In contrast, the flat and inclined PCHB configurations showed no significant changes in phycocyanin content. Similarly, the allophycocyanin content followed this pattern, with the highest (48.12 ± 1.5 mg/L) was found under the round-shaped PCHB C-PBR, while the lowest production (39.21 ± 1.1 mg/L) observed in microalgae cultivated under the control C-PBR, as shown in Fig. 5D. Introducing PCHB represents a significant breakthrough in enhancing both biomass and phycocyanin contents. Through spiral flow movement of the suspension an optimized light supply is promoted, which is especially advantageous for the cells in the central part of column. The PCHB baffles offer a scalable and practical solution for large-scale applications, providing an efficient alternative to fixed baffles. They are removable, easy to install, and maintain. The proposed C-PBR system with PCHB is economically feasible because nylon material is extensively utilized in 3D printing owing to its cost-efficiency, robustness, fracture resistance, and durability, rendering it a reliable and economical material. These characteristics facilitate scalability of the system, allowing easy up-scaling.

## Conclusions

To enhance CO_2_ fixation and mass transfer, various PCHBs were introduced into C-PBRs, which significantly promoted microalgal growth. Round-shaped PCHB increased mass transfer coefficient by 21%, yielding a higher dry mass (2.81 ± g/L) compared to flat, inclined PCHBs, and the control (1.57 g/L). Additionally, PCHB boosted microalgal pigment content in C-PBRs. The round-shaped PCHB increased chlorophyll-a content by 26% and phycocyanin content by 18% when compared with control C-PBR. Results verified that round-shaped PCHB generated stronger spiral vortices, producing smaller CO_2_ bubbles. The approach is highly promising for maximizing industrial C-PBRs, with the positive effect of efficiently advancing CO_2_ sequestration.

## Data Availability

No datasets were generated or analysed during the current study.

## References

[1] Mohsin M, Rasheed AK, Sun H, Zhang J, Iram R, Iqbal N, et al. Developing low carbon economies: an aggregated composite index based on carbon emissions. Sustain Energy Technol Assess. 2019;35:365–74.

[2] Li S, Chang H, Zhang S, Ho S-H. Production of sustainable biofuels from microalgae with CO2 bio-sequestration and life cycle assessment. Environ Res. 2023;227:115730. 36958384 10.1016/j.envres.2023.115730

[3] Mensah CN, Long X, Dauda L, Boamah KB, Salman M, Appiah-Twum F, et al. Technological innovation and green growth in the Organization for Economic Cooperation and Development economies. J Clean Prod. 2019;240:118204.

[4] Dasan YK, Lam MK, Yusup S, Lim JW, Show PL, Tan IS, et al. Cultivation of Chlorella vulgaris using sequential-flow bubble column photobioreactor: a stress-inducing strategy for lipid accumulation and carbon dioxide fixation. J CO Utiliz. 2020;41:101226.

[5] Hou C, Zhao J, Huang B, Zhou X, Zhang Y. Microalgae-based technologies for carbon neutralization and pollutant remediation: a comprehensive and systematic review. Resour Conserv Recycl. 2024;202: 107323.

[6] Udaypal, Goswami RK, Mehariya S, Verma P. Advances in microalgae-based carbon sequestration: Current status and future perspectives. Environ Res 2024;249:118397. 10.1016/j.envres.2024.118397 38309563

[7] Qin S, Wang K, Gao F, Ge B, Cui H, Li W. Biotechnologies for bulk production of microalgal biomass: from mass cultivation to dried biomass acquisition. Biotechnol Biofuels Bioprod. 2023;16:131. 10.1186/s13068-023-02382-4. 37644516 10.1186/s13068-023-02382-4 PMC10466707

[8] Kandasamy S, Zhang B, He Z, Bhuvanendran N, EL-Seesy AI, Wang Q, et al. Microalgae as a multipotential role in commercial applications: current scenario and future perspectives. Fuel. 2022;308:122053.

[9] Ravikumar Y, Razack SA, Yun J, Zhang G, Zabed HM, Qi X. Recent advances in Microalgae-based distillery wastewater treatment. Environ Technol Innov. 2021;24: 101839.

[10] Wagner H, Schad A, Höhmann S, Briol TA, Wilhelm C. Carbon and energy balance of biotechnological glycolate production from microalgae in a pre-industrial scale flat panel photobioreactor. Biotechnol Biofuels. 2024;17:42. 10.1186/s13068-024-02479-4. 10.1186/s13068-024-02479-4 PMC10941469 38486283

[11] Mathivanan K, Ameen F, Zhang R, Ravi G, Beduru S. Bubble column photobioreactor (BCPR) for cultivating microalgae and microalgal consortium (Co-CC) with additional CO2 supply for enhancing biomass, lipid, and preferable fatty acids production. Environ Res. 2023;238:117284. 37793593 10.1016/j.envres.2023.117284

[12] Bose A, Oshea R, Lin R, Murphy JD. Design, commissioning, and performance assessment of a lab-scale bubble column reactor for photosynthetic biogas upgrading with spirulina platensis. Ind Eng Chem Res. 2021;60:5688–704. 34276129 10.1021/acs.iecr.0c05974 PMC8277169

[13] Feng A, Zhang T, Li Y, Zhang H, Liu C. A study on the designs of baffles-enhanced gas transfer inside *Chlorella vulgaris* airlift photobioreactors and flow visualisation modelling. Sep Purif Technol. 2024;336: 126116.

[14] Raeisossadati M, Moheimani NR, Parlevliet D. Red and blue luminescent solar concentrators for increasing *Arthrospira platensis* biomass and phycocyanin productivity in outdoor raceway ponds. Bioresour Technol. 2019;291: 121801. 31326685 10.1016/j.biortech.2019.121801

[15] Murray AM, Fotidis IA, Isenschmid A, Haxthausen KRA, Angelidaki I. Wirelessly powered submerged-light illuminated photobioreactors for efficient microalgae cultivation. Algal Res. 2017;25:244–51.

[16] Feng A, Zhang T, Li Y, Zhang H, Liu C. A study on the designs of baffles-enhanced gas transfer inside *Chlorella vulgaris* airlift photobioreactors and flow visualisation modelling. Sep Purif Technol. 2024;336:126116.

[17] Ye Q, Cheng J, Guo W, Xu J, Li K, Zhou J. Serial lantern-shaped draft tube enhanced flashing light effect for improving CO2 fixation with microalgae in a gas-lift circumflux column photobioreactor. Bioresour Technol. 2018;255:156–62. 29414161 10.1016/j.biortech.2018.01.127

[18] Yang Z, Cheng J, Xu X, Zhou J, Cen K. Enhanced solution velocity between dark and light areas with horizontal tubes and triangular prism baffles to improve microalgal growth in a flat-panel photo-bioreactor. Bioresour Technol. 2016;211:519–26. 27038260 10.1016/j.biortech.2016.03.145

[19] Naira VR, Das D, Maiti SK. A novel bubble-driven internal mixer for improving productivities of algal biomass and biodiesel in a bubble-column photobioreactor under natural sunlight. Renew Energy. 2020;157:605–15.

[20] Cheng J, Xu J, Lu H, Ye Q, Liu J, Zhou J. Generating cycle flow between dark and light zones with double paddlewheels to improve microalgal growth in a flat plate photo-bioreactor. Bioresour Technol. 2018;261:151–7. 29656228 10.1016/j.biortech.2018.04.022

[21] Fu J, Huang Y, Liao Q, Zhu X, Xia A, Zhu X, et al. Boosting photo-biochemical conversion and carbon dioxide bio-fixation of *Chlorella vulgaris* in an optimized photobioreactor with airfoil-shaped deflectors. Bioresour Technol. 2021;337:125355. 34120064 10.1016/j.biortech.2021.125355

[22] Wang R, Li Z, Shi J, Holmes M, Wang X, Zhang J, et al. Color 3D printing of pulped yam utilizing a natural pH sensitive pigment. Addit Manuf. 2021;46: 102062.

[23] Ye Q, Cheng J, Guo W, Xu J, Li H, Zhou J. Numerical simulation on promoting light/dark cycle frequency to improve microalgae growth in photobioreactor with serial lantern-shaped draft tube. Bioresour Technol. 2018;266:89–96. 10.1016/j.biortech.2018.06.055. 29957295 10.1016/j.biortech.2018.06.055

[24] Guo X, Yao L, Huang Q. Aeration and mass transfer optimization in a rectangular airlift loop photobioreactor for the production of microalgae. Bioresour Technol. 2015;190:189–95. 25958141 10.1016/j.biortech.2015.04.077

[25] Manjrekar ON, Sun Y, He L, Tang YJ, Dudukovic MP. Hydrodynamics and mass transfer coefficients in a bubble column photo-bioreactor. Chem Eng Sci. 2017;168:55–66.

[26] Soni RA, Sudhakar K, Rana RS. Comparative study on the growth performance of Spirulina platensis on modifying culture media. Energy Rep. 2019;5:327–36.

[27] Song Y, Cheng J, Lai X, Guo W, Yang W. Developing a three-dimensional tangential swirl plate photobioreactor to enhance mass transfer and flashlight effect for microalgal CO2 fixation. Chem Eng Sci. 2021;244: 116837.

[28] Ye Q, Cheng J, Liu S, Qiu Y, Zhang Z, Guo W, et al. Improving light distribution and light/dark cycle of 900 L tangential spiral−flow column photobioreactors to promote CO2 fixation with Arthrospira sp. cells. Sci Total Environ. 2020;720:137611. 10.1016/j.scitotenv.2020.137611. 32325586 10.1016/j.scitotenv.2020.137611

[29] Ye Q, Cheng J, Lai X, An Y, Chu F, Zhou J, et al. Promoting photochemical efficiency of chlorella PY-ZU1 with enhanced velocity field and turbulent kinetics in a novel tangential spiral-flow column photobioreactor. ACS Sustain Chem Eng. 2019;7:384–93. 10.1021/acssuschemeng.8b03718.

[30] Cheng J, Guo W, Song Y, Kumar S, Ameer Ali K, Zhou J. Enhancing vorticity magnitude of turbulent flow to promote photochemical efficiency and trichome helix pitch of *Arthrospira platensis* in a raceway pond with conic baffles. Bioresour Technol. 2018;269:1–8. 10.1016/j.biortech.2018.08.058. 30144753 10.1016/j.biortech.2018.08.058

[31] Dong S, Jiang Y, Bu Y, Wang S, Zhang H, Wang R. Growth, photosynthetic pigment proteins responses and transcriptome analysis provide insights into survival strategies against short-term cold stress in the blue-green algae. Arthrospira Aquac Rep. 2022;27:101403.

[32] Kumar S, Ali Kubar A, Zhu F, Shao C, Cui Y, Hu X, et al. Sunlight filtered via translucent-colored polyvinyl chloride sheets enhanced the light absorption capacity and growth of Arthrospira platensis cultivated in a pilot-scale raceway pond. Bioresour Technol. 2023;386: 129501. 37468013 10.1016/j.biortech.2023.129501

[33] Manica R, Klaseboer E, Chan DYC. The hydrodynamics of bubble rise and impact with solid surfaces. Adv Colloid Interface Sci. 2016;235:214–32. 27378067 10.1016/j.cis.2016.06.010

[34] Sanaye Mozaffari Sabet N, Golzary A. CO2 biofixation at microalgae photobioreactors: hydrodynamics and mass transfer study. Int J Environ Sci Technol. 2022;19:11631–48.

[35] Cheng J, Song Y, Miao Y, Guo W, Wang Y, Li X, et al. Three-stage shear-serrated aerator broke CO2 bubbles to promote mass transfer and microalgal growth. ACS Sustain Chem Eng. 2020;8:939–47.

[36] Wang J, Hu C, He W, Du F, Jiao N, Liu J, et al. Novel thin-layer fountain photobioreactors for the high-density cultivation of Spirulina sp. ACS Sustain Chem Eng. 2023;11:16818–27.

[37] Rezvani F, Rostami K. Photobioreactors for utility-scale applications: effect of gas–liquid mass transfer coefficient and other critical parameters. Environ Sci Pollut Res. 2023;30:76263–82. 10.1007/s11356-023-27644-4. 10.1007/s11356-023-27644-4 37247144

[38] Kubar AA, Cheng J, Kumar S, Liu S, Chen S, Tian J. Strengthening mass transfer with the Tesla-valve baffles to increase the biomass yield of Arthrospira platensis in a column photobioreactor. Bioresour Technol. 2021;320: 124337. 33157436 10.1016/j.biortech.2020.124337

[39] Wang C, Lan CQ. Effects of shear stress on microalgae—a review. Biotechnol Adv. 2018;36:986–1002. 29524464 10.1016/j.biotechadv.2018.03.001

[40] Luna-Brito MJ, Sacramento-Rivero JC, Baz-Rodríguez SA. Effects of medium composition and gas superficial velocity on mass transfer during microalgae culturing in a bubble column photobioreactor. Ind Eng Chem Res. 2018;57:17058–63.

[41] Huang J, Feng F, Wan M, Ying J, Li Y, Qu X, et al. Improving performance of flat-plate photobioreactors by installation of novel internal mixers optimized with computational fluid dynamics. Bioresour Technol. 2015;182:151–9. 10.1016/j.biortech.2015.01.067. 25689309 10.1016/j.biortech.2015.01.067

[42] Fu J, Huang Y, Liao Q, Zhu X, Xia A, Zhu X, et al. Boosting photo-biochemical conversion and carbon dioxide bio-fixation of Chlorella vulgaris in an optimized photobioreactor with airfoil-shaped deflectors. Bioresour Technol. 2021;337:125355. 10.1016/j.biortech.2021.125355. 34120064 10.1016/j.biortech.2021.125355

[43] Yang Z, Cheng J, Xu X, Zhou J, Cen K. Enhanced solution velocity between dark and light areas with horizontal tubes and triangular prism baffles to improve microalgal growth in a flat-panel photo-bioreactor. Bioresour Technol. 2016;211:519–26. 10.1016/j.biortech.2016.03.145. 27038260 10.1016/j.biortech.2016.03.145

[44] Kumar S, Cheng J, Ali Kubar A, Guo W, Song Y, Liu S, et al. Orange light spectra filtered through transparent colored polyvinyl chloride sheet enhanced pigment content and growth of Arthrospira cells. Bioresour Technol. 2021;319: 124179. 33038649 10.1016/j.biortech.2020.124179

[45] Kubar AA, Kumar S, Liu W, Cui Y, Zhu F, Xu X, et al. Numerical simulation of vortex flow field generated in a novel nested-bottled photobioreactor to improve Arthrospira platensis growth. Bioresour Technol. 2023;373:128710. 36754237 10.1016/j.biortech.2023.128710

[46] Antonacci A, Lambreva MD, Margonelli A, Sobolev AP, Pastorelli S, Bertalan I, et al. Photosystem-II D1 protein mutants of Chlamydomonas reinhardtii in relation to metabolic rewiring and remodelling of H-bond network at QB site. Sci Rep [Internet]. 2018;8:14745. 10.1038/s41598-018-33146-y. 30283151 10.1038/s41598-018-33146-y PMC6170454

[47] Xu J, Cheng J, Xin K, Xu J, Yang W. Strengthening flash light effect with a pond-tubular hybrid photobioreactor to improve microalgal biomass yield. Bioresour Technol. 2020;318: 124079. 32911369 10.1016/j.biortech.2020.124079

[48] Kumar S, Cheng J, Jia D, Ali Kubar A, Yang W. Enhancing microalgae production by installing concave walls in plate photobioreactors. Bioresour Technol. 2022;345: 126479. 34864173 10.1016/j.biortech.2021.126479

[49] Luimstra VM, Schuurmans JM, Verschoor AM, Hellingwerf KJ, Huisman J, Matthijs HCP. Blue light reduces photosynthetic efficiency of cyanobacteria through an imbalance between photosystems I and II. Photosynth Res. 2018;138:177–89. 30027501 10.1007/s11120-018-0561-5 PMC6208612

[50] Pez Jaeschke D, Rocha Teixeira I, Damasceno Ferreira Marczak L, Domeneghini Mercali G. Phycocyanin from spirulina: a review of extraction methods and stability. Food Res Int. 2021. 10.1016/j.foodres.2021.110314 33992333

[51] McHardy C, Luzi G, Lindenberger C, Agudo JR, Delgado A, Rauh C. Numerical analysis of the effects of air on light distribution in a bubble column photobioreactor. Algal Res. 2018;31:311–25.

[52] Ye Q, Cheng J, Liu S, Qiu Y, Zhang Z, Guo W, et al. Improving light distribution and light/dark cycle of 900 L tangential spiral−flow column photobioreactors to promote CO2 fixation with Arthrospira sp. cells. Sci Total Environ. 2020;720:137611. 32325586 10.1016/j.scitotenv.2020.137611

